# Predicting herb-disease associations using network-based measures in human protein interactome

**DOI:** 10.1186/s12906-024-04503-4

**Published:** 2024-06-06

**Authors:** Seunghyun Wang, Hyun Chang Lee, Sunjae Lee

**Affiliations:** 1 Department of Bio and Brain Engineering, KAIST, 291 Daehak-ro, Yuseong-gu, Daejeon, 34141 Republic of Korea; 2 Division of Environmental Science and Ecological Engineering, Korea University, 145 Anam-ro, Seungbuk-gu, Seoul, 02841 Republic of Korea; 3 School of Life Sciences, GIST, 123 Cheomdan-gwagi-ro, Buk-gu, Gwangju, 61005 Republic of Korea

**Keywords:** Natural herb, Multi-compound multi-target (MCMT), Human protein interactome

## Abstract

**Background:**

Natural herbs are frequently used to treat diseases or to relieve symptoms in many countries. Moreover, as their safety has been proven for a long time, they are considered as main sources of new drug development. However, in many cases, the herbs are still prescribed relying on ancient records and/or traditional practices without scientific evidences. More importantly, the medicinal efficacy of the herbs has to be evaluated in the perspective of MCMT (multi-compound multi-target) effects, but most efforts focus on identifying and analyzing a single compound experimentally. To overcome these hurdles, computational approaches which are based on the scientific evidences and are able to handle the MCMT effects are needed to predict the herb-disease associations.

**Results:**

In this study, we proposed a network-based *in silico* method to predict the herb-disease associations. To this end, we devised a new network-based measure, WACP (weighted average closest path length), which not only quantifies proximity between herb-related genes and disease-related genes but also considers compound compositions of each herb. As a result, we confirmed that our method successfully predicts the herb-disease associations in the human protein interactome (AUROC = 0.777). In addition, we observed that our method is superior than the other simple network-based proximity measures (e.g. average shortest and closest path length). Additionally, we analyzed the associations between *Brassica oleracea var. italica* and its known associated diseases more specifically as case studies. Finally, based on the prediction results of the WACP, we suggested novel herb-disease pairs which are expected to have potential relations and their literature evidences.

**Conclusions:**

This method could be a promising solution to modernize the use of the natural herbs by providing the scientific evidences about the molecular associations between the herb-related genes targeted by multiple compounds and the disease-related genes in the human protein interactome.

## Backgrounds

Natural herbs, which is one of the main sources of traditional medicine, have been used for a long time to treat diseases or to relieve symptoms along with the history of mankind [1–3]. Recently, the herbs are frequently used as traditional, alternative and complementary medicine (TCAM). For example, more than 50% of the populations in East Asian countries (China, the Philippines, Republic of Korea) are visited the TCAM provider in last 12-month [4]. Not only in the eastern countries, but also in western countries, the traditional medicine is prevalently used. For instance, more than 80% of the populations are satisfied with the TCAM in Australia, Denmark, Slovenia, Spain, and Switzerland [4]. Moreover, the exports of the traditional medicine products from China to the United States and European countries amounted to $7.6 billion and $2 billion in 2010, respectively [3].

Meanwhile, as the safety of the natural herbs has been proven for a long time, they have been in the limelight as sources of new drug development. More than half of small molecule drugs approved by to the US Food and Drug Administration (FDA) between 1981 and 2019 are originated from the natural products [5]. Moreover, there are successful examples of modern medicine which are originated from the herbs [6]. For example, artemisinin is isolated from *Artemisia annua* which is the herb used in traditional Chinese medicine for hundreds of years and it has been used as one of leading antimalarial agents [7]. In addition, Arsenic trioxide, which is used as common ingredient of traditional Chinese medicine, is also approved by FDA for treatment of leukemia in 2000 [8].

Likewise, the role of the herbs is getting more important in drug development. However, the herb-based drug discovery faces some hurdles. First of all, the herbs are still prescribed relying on ancient records and traditional practices, not verified efficacy or molecular mechanisms based on scientific evidences [9]. More importantly, the most notable characteristic of the herbs is multi-compound multi-target (MCMT) effects, which refers that each herb contains multiple compounds and the compounds could target multiple proteins. It is considered one of great advantages of the herbs because the biological systems achieve robustness through redundancy [10–13] and targeting multiple disease genes could strength the medicinal efficacy by perturbing the systems, rather than individual disease genes [14]. However, most attempts of the herb-based drug discovery still rely on identifying and analyzing the most active single compound [6, 9]. In addition, there is no effective way to evaluate the medicinal efficacy of multiple compounds experimentally [6, 9].

To overcome these hurdles, systems pharmacology could be a promising solution. Systems pharmacology arose to overcome the limitations of traditional drug design paradigm known as ‘one gene, one drug, one disease’ and it analyzes the therapeutic effects of multiple target genes based on network analysis [14]. Therefore, it could be a great tool to understand how the multiple compounds in each herb affect the biological systems enabling the modern medicine to handle the MCMT effect [9, 15–17]. In practice, it has been reported that systems pharmacology could be applicated in predicting pharmacological targets of the herbs [18–21], predicting indications of the herbal compounds [22–24], and predicting synergistic combination of the herbs [25, 26].

In this study, we developed a network-based in silico method to predict the herb-disease associations. To this end, we devised a new network-based measure, WACP (weighted average closest path length), which not only quantifies proximity between herb-related genes and disease-related genes but also consider compound compositions of each herb. We evaluated a prediction performance of our method through AUROC score and we compared the prediction performance with the simple network-based proximity measures such as average shortest and closest path length. Besides the global approach which consider all herb-disease associations to evaluate the prediction performance, we measured the AUROC scores in individual herbs and diseases and explored the correlations between the AUROC scores and the number of known associated herbs or diseases. Additionally, we analyzed the associations between *Brassica oleracea var. italica* and its known associated diseases more specifically as case studies. Finally, based on the prediction results of the WACP, we suggested novel herb-disease pairs which are expected to have potential relations and their literature evidences.

## Methods

### Collecting disease-related genes and herb-related genes

**Fig. 1:**
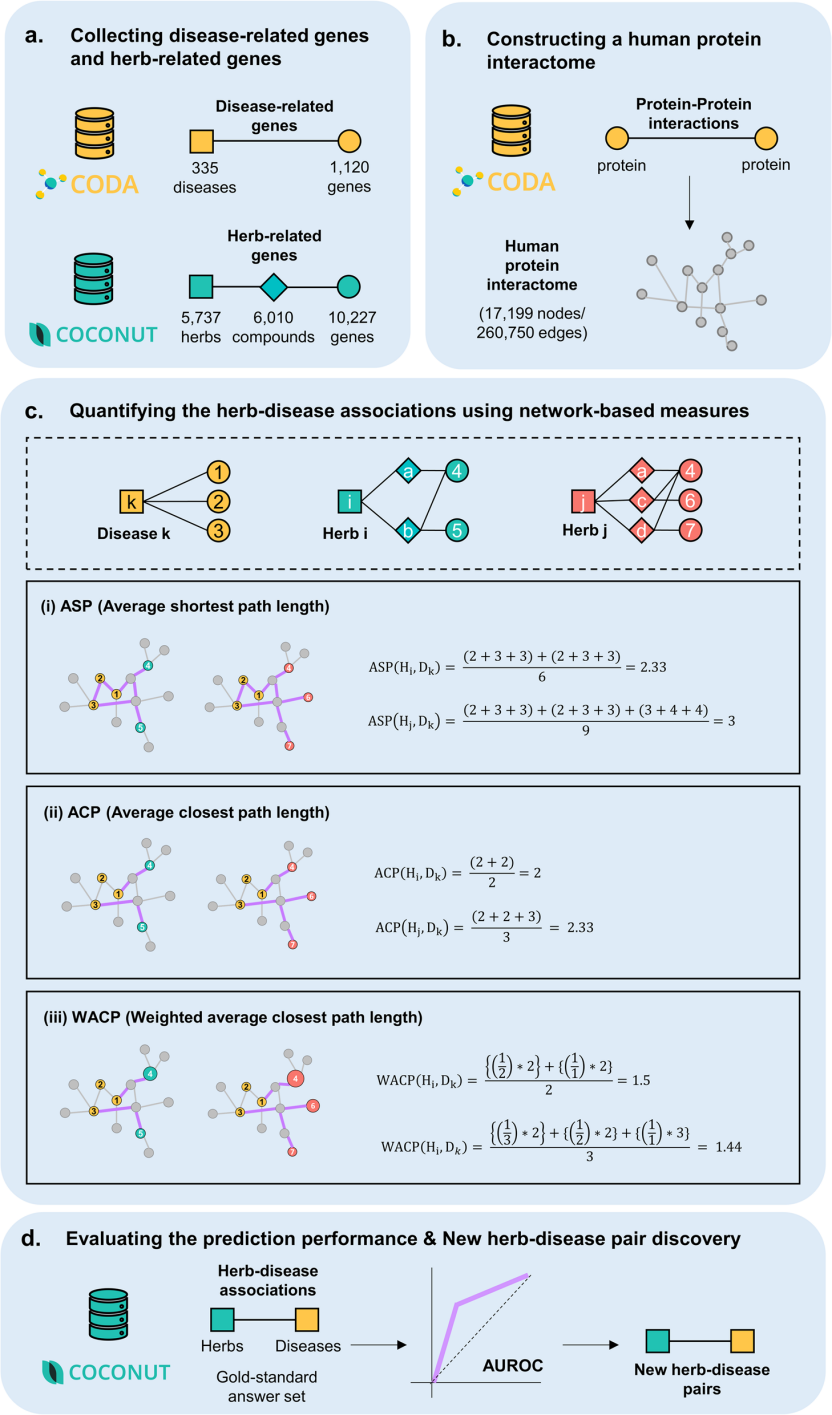
Method overview (**a**) We collected the disease-related genes of 335 diseases from the CODA repository and herb-related genes of 5,737 herbs based on the herb-compound and the compound-gene associations from the COCONUT database. **b** We constructed a human interactome network using 260,750 protein-protein interactions between 17,199 genes obtained from the CODA repository. **c** We quantified the herb-disease associations using three different network-based measures in the human protein interactome, including average shortest and closest path length and weighted closest path length that we devised in this study. **d** We evaluated the performance of each network-based measure through AUROC scores using the known herb-disease pairs in the COCONUT database as gold-standard

We used CODA (Context-Oriented Directed Association) repository [27] to collect disease-related genes (Fig. 1a). Briefly, CODA is the repository that integrates biological associations of both molecular level entities and phenomic level entities with anatomical context. Among the various types of associations in the CODA repository, we collected 163,212 disease-gene associations of 3,467 diseases and 15,647 genes from the CODA repository, which are obtained from multiple databases such as CTD [28], DiseaseConnect [29] and EndoNet [30]. To obtain more reliable disease-gene associations, we manually chose the disease-gene associations that have the evidences in at least two databases and resulted in the 2,098 disease-gene associations within 335 diseases and 1,120 genes.

To collect herb-related genes, we used COCONUT database (Compound Combination-Oriented Natural Product Database with Unified Terminology) [31] (Fig. 1a). Briefly, the COCONUT is an integrated database of comprehensive information about natural products with unified and standardized terminology. We estimated the herb-related genes through compounds in each herb and their target genes. The COCONUT database contains 1,138,081 herb-compound associations between 15,980 herbs and 52,453 compounds obtained from TCMID [32], KTKP (https://www.koreantk.com/ktkp2014/), TCM-ID [33], HerDing [34], CMAUP [35], NPASS [36], and FooDB (https://foodb.ca/). Among them, we chose the 317,051 herb-compound associations between 6,339 herbs and 26,342 compounds which have the evidences in at least two databases.

Among the 26,342 compounds, we chose the 6,010 compounds which have at least one functional and/or physical target genes based on compound-gene associations existed in the COCONUT databases, which are obtained from the MATADOR [37], BindingDB [38], STITCH [39], ChEMBL [40], CTD [28], DCDB [41], and DrugBank [42] and the databases that we mentioned above for the herb-compound associations. Like other associations, we chose the compound-gene associations which have the evidence in at least two databases and it resulted in 100,847 compound-gene associations between 6,010 compounds and 10,227 genes and we finally selected 5,737 herbs that contain at least one compound among these 6,010 compounds.

### Constructing a human interactome network

We constructed human protein interactome using protein-protein interactions from the CODA repository [27]. The CODA repository compiled the protein-protein interactions from several databases including BioGRID [43], KEGG [44] and EndoNet [30]. The 260,770 protein-protein interactions between 17,224 proteins exist in the CODA repository, and we used the largest connected components of the interactome for the following analysis, which is consisted of the 260,750 interactions between 17,199 proteins (Fig. 1b).

### Quantifying the herb-disease associations using network-based measures

For each herb *i* and disease *k*, we defined herb-related genes ($${H}_{i})$$ as the union set of the target genes of the compounds contained in the corresponding herb and disease-related genes ($${D}_{k})$$as the set of disease genes associated with the corresponding disease. When the herb *i* has *N* herb-related genes and the disease *k* has *M* disease-related genes, we defined each herb-related gene in $${H}_{i}$$ as $${h}_{in}$$ and each disease-related gene in $${D}_{k}$$ as $${d}_{km}$$, respectively. Given this, we used three different network-based measures to quantify the associations between the 5,737 herbs and 335 diseases; (i) ASP (average shortest path length), (ii) ACP (average closest path length), and (iii) WACP (weighted average closest path length).

(i) The average shortest path length (ASP) is one of the most commonly used measures to quantify the proximity between nodes in networks [45]. In this study, we hypothesized that the more closely the herb-related genes locate to the disease-related genes, the stronger associations exist. Therefore, we measured the shortest path lengths between all herb-related genes and disease-related genes ($$\text{s}\text{p}\text{l}({h}_{in}$$, $${d}_{km})$$) in each herb-disease pair and averaged it (Eq. 1). For example, the ASP between disease *k* and herb *i* shorter than that between disease *k* and herb *j* because the gene 7 related to the herb j ($${h}_{j7}$$) is located farther from the three disease related genes ($${d}_{k1}, {d}_{k2}, {d}_{k3}$$) and herb *i* is predicted as more associated with disease *k* (Fig. 1c-(i)).
(ii) We also used the average closest path length (ACP) based on the hypothesis that each herb-related gene does not have to target all disease-related genes [46]. Therefore, it was defined as the averaged shortest path length between the herb-related genes and their closest disease-related genes (Eq. 2). For example, like ASP, the herb *i* is predicated as more associated with disease *k* than herb j in this measure because the gene 7 related to the herb *j* ($${h}_{j7}$$) has longer closest path length, $$\text{s}\text{p}\text{l}({h}_{j7}$$, $${d}_{k3})=3$$ (Fig. 1c-(ii)).
(iii) In addition to simple ASP and ACP, we hypothesized that the more compounds perturb the target genes, the more associations will be. Therefore, we devised the weighted average closest path length (WACP) to consider the compound compositions of the herbs. The inversed value of weight (${ w}_{in}$) which was defined as the number of compounds targeting each target gene (${h}_{in}$) in each herb is multiplied to the closest path length (Eq. 3) between the herb-related genes and disease-related genes in each herb-disease pair. We used inversed value of weight to coincide with the values of shortest path length of which smaller values indicate the closer associations. Unlike ASP and ACP, herb *j* is predicted as more associated with disease *k* than herb *i* because the gene 4 is perturbed by a greater number of compounds in the herb *j* (${w}_{j4}=3$) than the herb *i* (${w}_{i4}=2$) (Fig. 1c-(iii)).

Likewise, the association of each herb-disease pair can be differed by which network-based measure is used and we quantified the associations of all herb-disease pairs using each measure. We used python library *networkx* (version 2.6.3) for overall network analysis.

1 $$\text{A}\text{S}\text{P}\left({H}_{i}, {D}_{k}\right)= \frac{\sum _{i=1}^{N}\sum _{k=1}^{M}spl({h}_{in}, {d}_{km})}{N \times M}$$

2 $$\text{A}\text{C}\text{P}\left({H}_{i}, {D}_{k}\right)= \frac{\sum _{i=1}^{N}{min}_{m\in M}spl({h}_{in}, {d}_{km})}{N}$$

3 $$\text{W}\text{A}\text{C}\text{P}\left({H}_{i}, {D}_{k}\right)= \frac{\sum _{i=1}^{N}\left(\frac{1}{{w}_{in}}\right)*{(min}_{m\in M}spl({h}_{in}, {d}_{km}))}{N}$$

### Evaluating prediction performance

To evaluate the prediction performance of each network-based measures (Fig. 1d), we obtained 12,208 herb-disease associations between the herbs and the diseases from COCONUT database [31], which are obtained from public databases, such as TCMID [32], TCM-ID [33], KTKP (https://www.koreantk.com/ktkp2014/), BFN (https://biofood.or.kr), in-house text-mining and experiments. The public databases mainly collected the herb-disease associations from the reputable traditional Chinese or Korean medicine books and the publications through text mining methods. These pairs are known as that each herb has therapeutic effects on the corresponding diseases and we used them as gold-standard answer set (Fig. 1d). To measure AUROC score, we ranked all herb-disease pair based on the values in each of network-based measures and regarded the herb-disease pair as true positives if they exist in the gold-standard answer set. We used python *scikit-learn* (version 1.1.1) for calculating the AUROC scores.

## Results

### Predicting the herb-disease associations

**Fig. 2:**
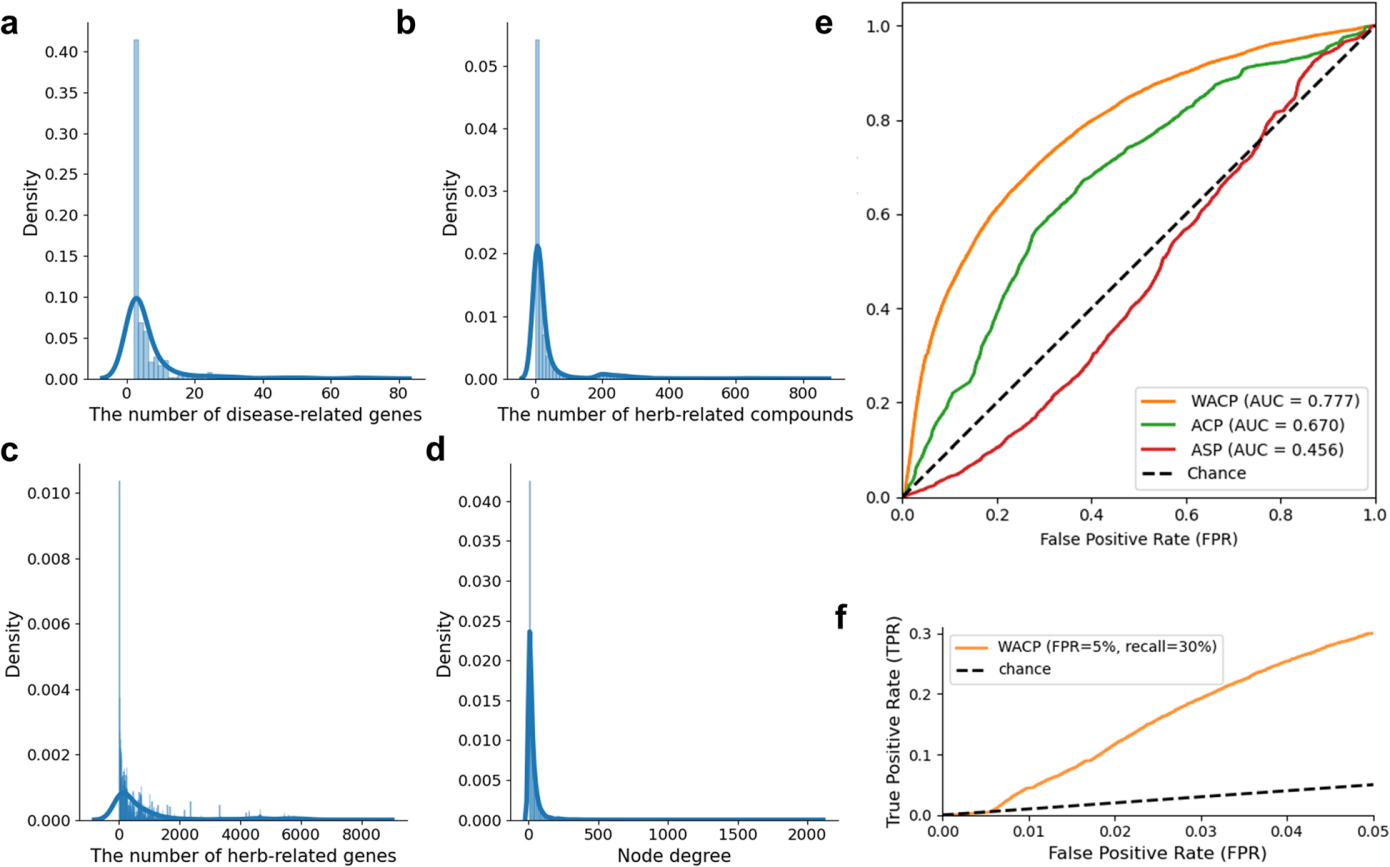
The statistics of the disease-related genes, the herb-related genes and the human protein interactome and the prediction performance of each network-based measures (AUROC). **a** The distribution of the number of related genes in the 335 diseases (**b**) The distribution of the number of associated compounds in the 5,737 herbs, **c** The distribution of the number of related genes in the 5,737 herbs (**d**) The distribution of the node degrees in the human protein interactome, **e** The ROC curve of each network-based proximity measures and (f) The ROC curve of the WACP at highly conservative false-positive rate (FPR = 5%)

First of all, we explored the statistics of the disease-related genes, the herb-related genes, and the human protein interactome which we collected from the CODA repository and the COCONUT database (Fig. 1a and b). The 335 diseases had 6.26 related genes on average (Fig. 2a). Each disease had at least two related genes and a maximum of 74 related genes. Meanwhile, the 5,737 herbs contained 34.50 associated compounds on average and each of herb contained a minimum of 1 and a maximum of 838 associated compounds (Fig. 2b). In addition, we estimated the herb-related genes through the genes that targeted by each compound and the herbs had 924.39 related genes on average and each of herb had a minimum of 1 and a maximum of 8,189 related genes (Fig. 2c). Furthermore, we constructed the human protein interactome consisted of 260,770 protein-protein interactions between 17,224 proteins. The average degree of the nodes was 30.32 and the minimum and maximum degree was 1 and 2,088 respectively (Fig. 2d). These results indicate that most of diseases and herbs are associated with more than one gene and the associations between them have to be analyzed more comprehensively through network analysis, rather than single gene-based approaches.

More importantly, to predict the herb-disease associations in the human protein interactome, we devised a new network-based measure named weighted average closest path length (WACP) which weights the herb-related genes by the number of compounds targeting each of them (Methods). We measured the WACP of all herb-disease pairs between 335 diseases and 5,737 herbs. The lower WACP value indicates the stronger associations and we ranked all herb-disease pairs based on their WACP values. Then, we evaluated the prediction performance of the WACP through AUROC (area under the receiver operating characteristic) using the known herb-disease associations obtained from the COCONUT database as a gold-standard answer set (Fig. 1d). Additionally, we measure the proximity between all herb-disease pairs using average shortest path length (ASP) and average closest path length (ACP) which are the most frequently used network-based proximity measure to compare the prediction performance.

As a result, the WACP (AUROC = 0.777) was superior to the ASP (AUROC = 0.456) and the ACP (AUROC = 0.670) (Fig. 2e). The WACP also show improved AURPC scores (0.023) than the baseline AUPRC scores (12,208 positive herb-disease pairs/1,921,225 all herb-disease pairs = 0.006), the ASP (AUPRC = 0.005) and the ACP (AUPRC = 0.011). This result indicates that considering the compound composition of each herb can improve the performance in the prediction of herb-disease associations in the human protein interactome, compared to the simple network-based measures that only consider the proximity between the genes. More specifically, the large number of true positive pairs in the gold-standard answer set were recovered at highly conservative false-positive rate (FPR = 5%, recall = 30%) (Fig. 2f). This prediction performance is notable because correct, but not discovered yet, predictions would be considered false positive and it can significantly underestimate the prediction performance. Taken together, we confirmed that the WACP successfully predict the herb-disease associations in the human protein interactome and they show better prediction performance than the other network-based proximity measures.

### Prediction performances in individual herbs and diseases

**Fig. 3:**
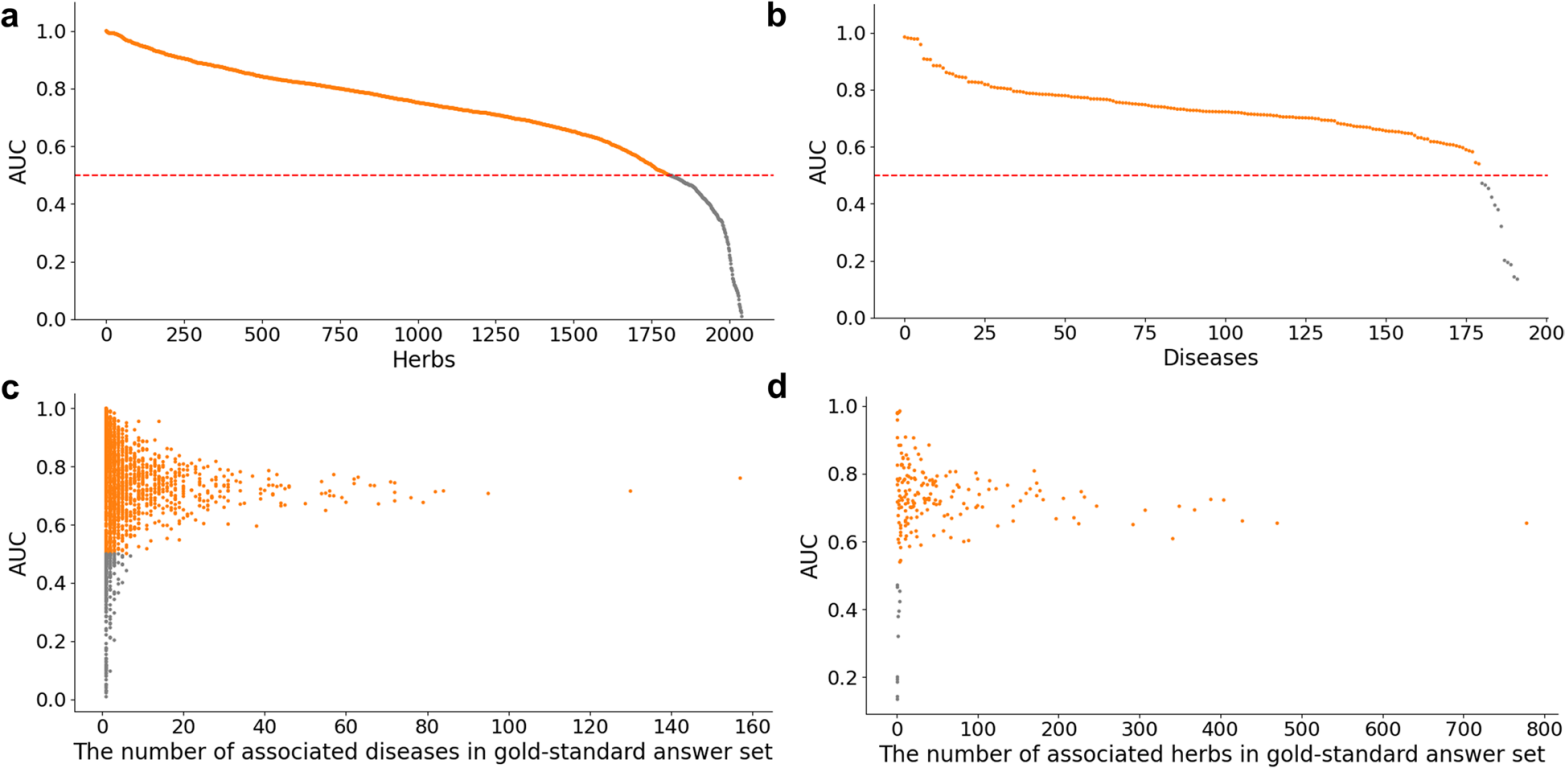
The distribution of AUROC scores in individual herbs and diseases. The AUROC scores of individual (**a**) herbs and (**b**) diseases are plotted using scatter plots. Each dot indicates the individual herbs and diseases and those that show higher AUROC scores than 0.5 were colored as orange. **c** The correlation between the AUROC scores of individual herbs and the number of their known associated diseases. (d) The correlation between the AUROC scores of individual disease and the number of their known associated herbs

Besides the global approach that use all herb-disease pairs to evaluate the prediction performance, we measured the AUROC scores in individual herbs and diseases. Among the 5,737 herbs and the 335 diseases, we selected the 2,041 herbs and the 192 diseases which have at least one associated disease and herb, respectively and we calculated AUROC scores in each herb and disease based on the WACP. As a result, we confirmed that the average AUROC scores of individual herbs and disease show similar prediction performance with the global approach, 0.721 (Fig. 3a) and 0.711 (Fig. 3b), respectively. It was notable that 88.4% and 93.8% of the 2,041 herbs and the 192 diseases showed the better performance than random (AUROC = 0.5).

Furthermore, we explored whether the prediction performances of individual herbs and diseases are affected by the number of known disease and herb pairs. To this end, we measured Pearson correlation coefficients (PCC) between the AUROC scores of individual herbs and diseases and the number of known disease and herb pairs. As shown in Fig. 3c, we confirmed that there is no significant correlation between the AUROC scores of individual herbs and the number of their known associated diseases (PCC = 0.011, *p*-value = 0.615). Similarly, there is no significant correlation between the AUROC scores of individual diseases and the number of their known associated herbs (PCC=-0.011, *p*-value = 0.881) (Fig. 3d).

### Case study: *Brassica oleracea var. italica*

For case study, we the selected reliable herbs that show the AUROC scores larger than 0.9 and have at least three known associated diseases. In addition, we found that the threshold of WACP at highly conservative false-positive rate (FPR = 5%) is 1.50 (Fig. 2f). Therefore, we again selected the herbs of which all WACP with their known associated diseases are lower than the threshold. This resulted in four herbs: *Gossypium*, *Glehnia littoralis, Citrus aurantiifolia*, and *Brassica oleracea var. italica* (Table 1). Among them, we chose *Brassica oleracea var. italica* which has the largest number of known associated diseases (*Malignant neoplasm of breast, Malignant neoplasm of prostate, Colorectal carcinoma* and *Cataract)* for the case study.

**Table 1:** The known herb-disease pairs of the herbs that show reliable prediction performances

	Herb	Disease	WACP	AUROC
1	*Gossypium*	*Breast Carcinoma*	1.188	0.983
2		*Melanoma*	1.266	
3		*Alzheimer’s disease*	1.299	
4	*Glehnia littoralis*	*Breast Carcinoma*	1.205	0.959
5		*Lung Adenocarcinoma*	1.408	
6		*Obesity*	1.410	
7	*Citrus aurantiifolia*	*Breast Carcinoma*	1.177	0.937
8		*Obesity*	1.393	
9		*Multiple Sclerosis*	1.416	
10	*Brassica oleracea var. italica*	*Malignant neoplasm of breast*	1.144	0.923
11		*Malignant neoplasm of prostate*	1.213	
12		*Colorectal carcinoma*	1.296	
13		*Cataract*	1.469	

**Fig. 4:**
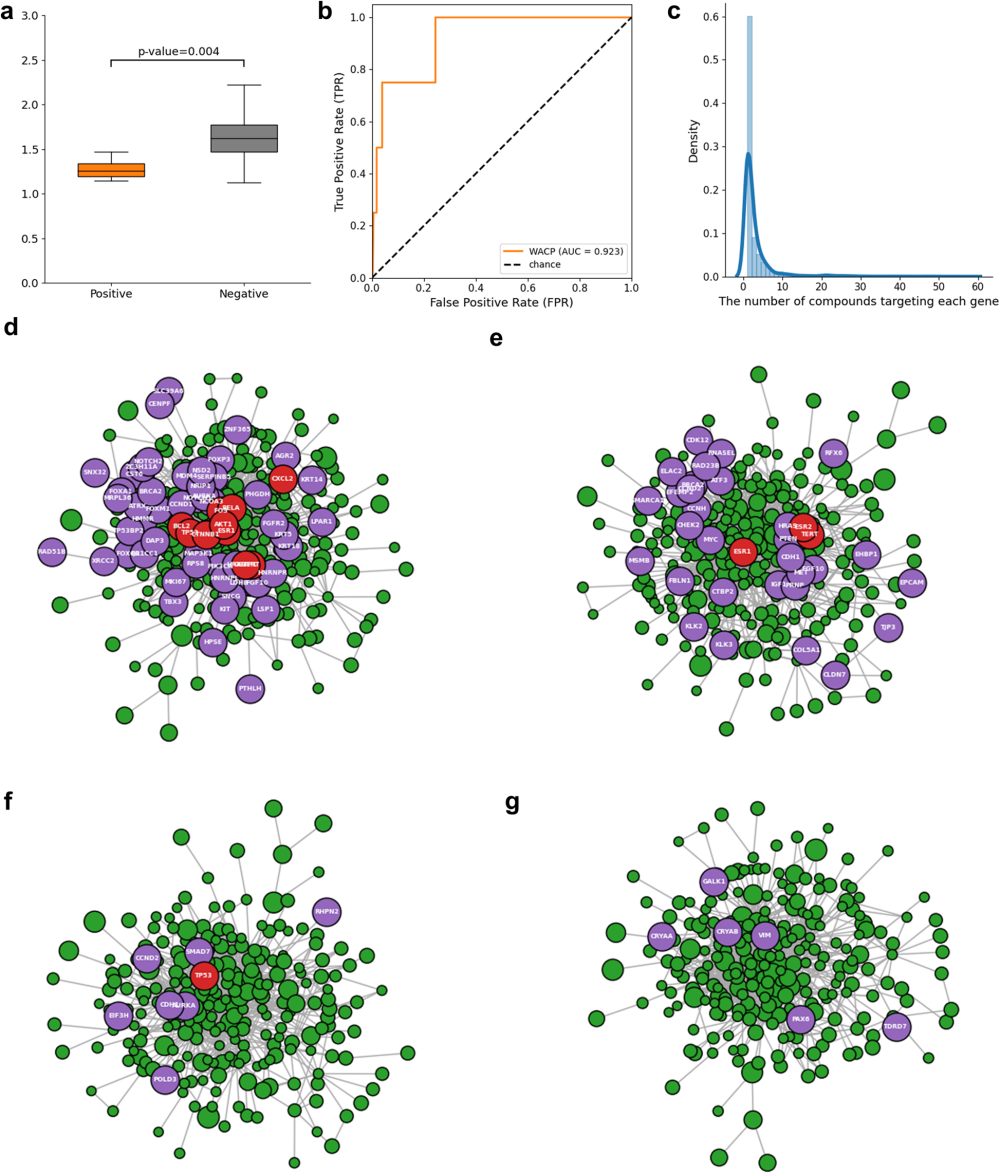
*Brassica oleracea var. italica* for case study (**a**) The WACP distributions of the known associated diseases and the other diseases were plotted using box plots. **b** The ROC curve of *Brassica oleracea var. italica.* (**c**) The distribution of the number of compounds targeting each gene related to *Brassica oleracea var. italica.* The network plot of the genes related with *Brassica oleracea var. italica* and the genes related with (**d**) *Malignant neoplasm of breast*, **e** *Malignant neoplasm of prostate*, **f** *Colorectal carcinoma* and (**g**) *Cataract.* To avoid overcrowding, we include only top 5% genes related to *Brassica oleracea var. italica* according to their weight values in the WACP. They are colored as green and the node size indicates the weights. The disease related gens are colored as purple and the nodes in the intersections are colored as red

More specifically, the four known associated diseases with *Brassica oleracea var. italica* had significantly lower WACP values than the other diseases (Rank-sum test, p-value = 0.004) (Fig. 4a) and its AUROC score was 0.923 (Table 1; Fig. 4b). Meanwhile, it contains 324 associated compounds and these compounds are related with 6,212 genes. Each gene related to *Brassica oleracea var. italica* is targeted by 3.14 compounds on average and the genes with high weights are targeted by at least 10 compounds (top 5%) (Fig. 4c).

It showed the lowest WACP values with *Malignant neoplasm of breast* and we observed that the genes with the high weights (top 5%), which means that they are targeted by many compounds contained in *Brassica oleracea var. italica*, are also the related genes of *Malignant neoplasm of breast*, such as *AKT1, BCL2, CTNNB1, CXCL2*, *ESR1, FOS, GSTP1 RELA*, *TERT and TP53* (Fig. 4d). Similarly, we confirmed that the genes related to *Malignant neoplasm of prostate* (e.g. *ESR1, ERS2* and *TP53*) (Fig. 4e) and *Colorectal carcinoma (e.g. TP53)* (Fig. 4f) are directly targeted by many compounds in *Brassica oleracea var. italica.* Interestingly, even though there are no genes that are directly targeted by the genes related to *Brassica oleracea var. italica*, the WACP successfully discovered *Cataract* as the associated disease (Fig. 4g).

### New herb-disease association discovery

Based on the reliable prediction performances of the WACP, we suggested new herb-disease associations which are expected to have potential relations. To this end, we focused on the four herbs that we discovered in the previous section. For the discovery of new herb-disease association, we selected the diseases that show the lower WACP than the average WACP of the known associated diseases in each herb. In addition, among them, we finally selected the disease of which AUROC score is better than the average AUROC score of individual disease (average AUROC = 0.711, Fig. 3b). The new herb-disease associations are presented in Table 2.

**Table 2:** Potential herb-phenotype associations of top 5 herbs showing highest AUROC scores

	Herb	Disease	WACP	Reference
1	*Glehnia littoralis*	*Prostatic Neoplasms*	1.265	[52]
2		*Skin Neoplasms*	1.292	[52, 53]
3		*Renal cell carcinoma*	1.333	[52]
4	*Citrus aurantifolia*	*Lung Neoplasms*	1.243	[54–57]
5		*Prostatic Neoplasms*	1.251	[54, 57]
6		*Skin Neoplasms*	1.274	[54]
7		*Renal cell carcinoma*	1.321	[54]
8	*Brassica oleracea var. italica*	*Lung Neoplasm*	1.204	[47–49]
9		*Skin Neoplasms*	1.238	[47, 50, 51]

For example, *Brassica oleracea var. italica* that is used for the case study in the previous section is expected to have potential associations on *Lung neoplasm* and *skin neoplasm.* Notably, the anti-cancer activity of *Brassica oleracea var. italica* has been reported in many studies [47], especially for *Lung neoplasm* [48, 49] and *skin neoplasm* [50, 51]. Similarly, we found literature evidences of the new herb-disease associations that we suggested in Table 2 and these pairs can be considered as new indications of the herbs along with follow-up studies.

## Discussion

Based on the prediction results of the WACP, we suggested the new herb-disease pairs which are expected to have potential associations and we found that most of them have literature evidences about their therapeutic effects. Even though all our suggestions were associated with the cancer, this might be resulted from a current hurdle of network biology research field that the prior knowledges are highly biased to the most actively studied diseases such as cancer [58]. If other diseases are further studied with the great manpower and research funding like cancer, the prior knowledge about herb- and disease-related genes could be complemented and our method could find new associations between the herbs and more various diseases.

Furthermore, beyond discovering the herb-disease associations, it is our priority to discriminate agonistic or antagonistic effects of the herbs against the diseases in the near future and the use of activation/inhibition information between the compounds and genes could be a starting point. In addition, each gene could have tissue-specific or cell type-specific interactions with other genes. For example, some transcription factors induce expression of certain genes only in the specific tissues [59]. Hence, applying tissue-specific or cell type-specific interactome which is related to the disease pathology enables our method to more precisely predict the herb-disease associations. Lastly, we used the herb-disease associations obtained from the public databases and the text-mining tool as gold-standard answer set. More precisely curated herb-disease associations (e.g., herb-disease associations extracted from the text mining tool and validated through *in-vitro* and *in-vivo* experiment) could increase the reliability of our method.

## Conclusions

In this study, we devised the new network-based network proximity measure named as WACP, which is the average closest path length between the disease-related genes and the herb-related genes which are weighted by the number of compounds targeting them in each herb. We demonstrated WACP is superior than the simple network-based proximity measures in the herb-phenotype association prediction. These results indicate that considering not only the proximity between the herb-related genes and the disease-related genes but also the compound compositions of each herb can improve the performance in the herb-disease association predictions in the human protein interactome.

In conclusion, we hope that our method could be a promising solution to modernize the use of the natural herbs by providing the scientific evidences through the molecular associations between the herb-related genes targeted by multiple compounds in each herb and the disease-related genes in the human protein interactome.

## Data Availability

The datasets used and/or analyzed during the current study are available from the corresponding author on reasonable request.

## Abbreviations

ASP: Average shortest path length
ACP: Average closest path length
WACP: Weighted closest path length
AUROC: Area under the receiver operating characteristic
